# Biochemical Pathways of Neuroplasticity in Sport Skill Acquisition: From Neuroscience to Coaching Practice

**DOI:** 10.3390/brainsci16070694

**Published:** 2026-06-30

**Authors:** Patrizia Proia, Alessandro Sclafani, Andrea Pagliaro, Anna Alioto, Alessia Boatta, Sara Baldassano, Giuseppe Messina, Erika Loi, Cristina Cortis, Armando Sangiorgio, Alessandra Amato

**Affiliations:** 1 Sport and Exercise Sciences Research Unit, Department of Psychology, Educational Science and Human Movement, University of Palermo, 90128 Palermo, Italy; patrizia.proia@unipa.it (P.P.); andrea.pagliaro@unipa.it (A.P.); anna.alioto@unipa.it (A.A.); alessandra.amato@unict.it (A.A.); 2 Department of Physical Education and Sports, Faculty of Sport Sciences, Sport and Health University Research Institute (iMUDS), University of Granada, 18016 Granada, Spain; alessandro.sclafani95@gmail.com; 3 Department of Human Sciences and Promotion of the Quality of Life, San Raffaele University, 00166 Rome, Italy; giuseppe.messina@uniroma5.it; 4 Institute for Biomedical Research and Innovation, National Research Council (CNR), 90146 Palermo, Italy; 5 Department of Biological, Chemical and Pharmaceutical Sciences and Technologies, University of Palermo, 90128 Palermo, Italy; sara.baldassano@unipa.it; 6 Department of Clinical and Experimental Sciences, University of Brescia, 25123 Brescia, Italy; erika.loi@unibs.it; 7 Department of Human Sciences, Society and Health, University of Cassino and Lazio Meridionale, 03043 Cassino, Italy; c.cortis@unicas.it; 8 Dipartimento di Scienze Umanistiche, Università Telematica Pegaso, Isola F2, Via Porzio, 80143 Napoli, Italy; dinosangiorgio@gmail.com

**Keywords:** motor learning, cerebellar plasticity, basal ganglia, primary motor cortex, error-based learning, reinforcement learning, use-dependent learning, cortical inhibition, elite athletes, skill automatization, BDNF, dopamine, glutamatergic plasticity, muscle–brain communication, neurovascular coupling

## Abstract

**Highlights:**

**What are the main findings?**

- Motor skill acquisition in sport emerges from the dynamic interaction of error-based, reinforcement, and use-dependent learning mechanisms.
- The relative contribution of cerebellar, basal ganglia, and motor cortex changes across the cognitive, associative, and autonomous phases of skill acquisition.
- Sport-induced neuroplasticity is supported by stage-dependent biochemical pathways, including cerebellar LTD, dopaminergic corticostriatal plasticity, glutamatergic LTP-like mechanisms, BDNF–TrkB signaling, and muscle–brain communication.

**What are the implications of the main findings?**

- Coaching strategies should be adapted to the learning phase, with explicit error-focused feedback in early learning and more outcome-based, context-specific practice in later stages.
- Sport-specific and environment-dependent neuroplasticity suggests that athletes should train in performance-relevant contexts to optimize automatization and skill robustness.

**Abstract:**

Background/Aim: Motor skill acquisition is the foundation of athletic performance, from the novice learning a new technique to the elite athlete executing complex movements automatically under pressure. Although classical models have defined the neural substrates of motor control—the cerebellum for error correction, the basal ganglia for action selection, and the primary motor cortex (M1) for execution—emerging evidence suggests that motor learning is the result of the dynamic interaction of multiple parallel processes rather than a linear hierarchy. This narrative review integrates classical neuroanatomical knowledge with contemporary findings on multisite plasticity, with a particular focus on sport-specific adaptations. Methods: We examined three core learning mechanisms operating in parallel: error-based learning (cerebellar-dependent, driven by sensory prediction errors), reinforcement learning (striatal-dependent, driven by reward prediction errors and dopamine), and use-dependent learning (cortical-dependent, driven by mere repetition). We also summarize the biochemical pathways supporting these learning processes, including glutamatergic LTP-like cortical plasticity, cerebellar mGluR1–PKC–LTD signaling, dopaminergic corticostriatal plasticity, BDNF–TrkB-dependent neurotrophic mechanisms, growth-factor signaling, and exercise-induced muscle–brain communication. Results: We then propose a spatiotemporal model in which the relative contribution of each network shifts dynamically across the three stages of skill acquisition, from the early cognitive/strategic phase to the late automatic phase characteristic of elite performance. At the molecular level, these stage-dependent adaptations are supported by synaptic strengthening and weakening mechanisms, reward-dependent dopamine signaling, neurotrophic and growth-factor-mediated remodeling, and peripheral metabolic/myokine signals that modulate brain plasticity during training and recovery. Special attention is given to contextual and sport-specific adaptations, using the paradigmatic example of elite swimmers who demonstrate enhanced short-interval intracortical inhibition (SICI) selectively in the aquatic environment, reflecting long-term sport-induced neuroplasticity. Conclusions: Understanding these dynamic network mechanisms has direct implications for coaching, training periodization, and the development of targeted neuromodulatory interventions to accelerate skill acquisition and optimize athletic performance.

## 1. Introduction: From the Static Homunculus to the Dynamic Athletic Brain

The ability to learn and execute complex movements is what sets elite athletes apart from beginners. Whether it is a gymnast performing a flawless floor routine, a tennis player executing a precise serve under pressure, or a basketball player controlling the ball without looking while dribbling, these performances are the result of countless hours of training that have reshaped the athlete’s brain [1]. But how does the nervous system transform a difficult and laborious movement into an automatic, efficient, and robust athletic skill?

For decades, our understanding of motor control has been shaped by classical neuroanatomical models: the motor homunculus of Penfield, the pyramidal and extrapyramidal tracts, and the functional specialization of the cerebellum (error correction), basal ganglia (action selection), and primary motor cortex (execution) [2,3,4,5]. These foundational contributions provided an indispensable framework. However, as recently articulated [6,7], this hierarchical and compartmentalized view is increasingly recognized as incomplete. The central challenge lies not in understanding each component in isolation but in deciphering how these components dynamically cooperate and compete across different phases of learning [8,9].

Critically for the sport scientist, motor learning is not a linear transfer of control from one brain region to another. Rather, it is a continuous, parallel, and weighted interplay of multiple learning systems operating across various spatial and temporal scales [10,11]. Moreover, the athlete’s brain exhibits contextual and sport-specific plasticity: neural adaptations that are tuned to the specific environment in which training occurs [12,13].

This narrative review has three aims, progressively oriented toward sport applications. First, we briefly revisit the classical neuroanatomical substrates of motor control to establish a common language for sport scientists. Second, we synthesize contemporary evidence on three fundamental learning processes: error-based, reinforcement, and use-dependent learning, and show how they operate in parallel during athletic training. Third and most importantly, we propose an integrated, spatiotemporal model of network reorganization and illustrate its implications for coaching, training periodization, and the optimization of sport performance.

## 2. Methodological Approach

This narrative review aimed to integrate classical neuroanatomical knowledge with contemporary evidence on the neurophysiological and biochemical mechanisms underlying sport skill acquisition, with particular emphasis on error-based learning, reinforcement learning, use-dependent plasticity, and sport-specific neuroplastic adaptations. To retrieve eligible articles, a manual search was conducted on the following databases: PubMed, Google Scholar, Scopus, and Web of Science. Articles published up to 31 December 2025 were considered, with no lower date limit. The search combined terms such as: “motor learning” OR “motor skill acquisition” OR “sensorimotor learning” OR “sport skill acquisition” AND “neuroplasticity” OR “cortical plasticity” OR “cerebellar plasticity” OR “striatal plasticity” AND “sport” OR “athlete” OR “exercise training” OR “coaching” AND “error-based learning” OR “reinforcement learning” OR “use-dependent learning” OR “cerebellum” OR “basal ganglia” OR “primary motor cortex” OR “dopamine” OR “BDNF” OR “glutamate” OR “GABA” OR “LTP” OR “LTD”. This research was extended by screening the reference lists of the selected articles. Articles were excluded when they were not written in English, were not directly relevant to motor learning or sport-related neuroplasticity, focused exclusively on non-motor cognitive processes, or did not provide mechanistic, neurophysiological, biochemical, or applied information relevant to the aims of the review. Two authors (PP and AA) conducted the literature search and ultimately resolved disagreements about article inclusion through discussion with a third researcher (AB). To identify duplicate studies, selected manuscripts from each database were loaded into Mendeley (Mendeley Cite v1.69.3).

## 3. Classical Substrates of Motor Control: A Foundation for Sport

**Figure 1:**
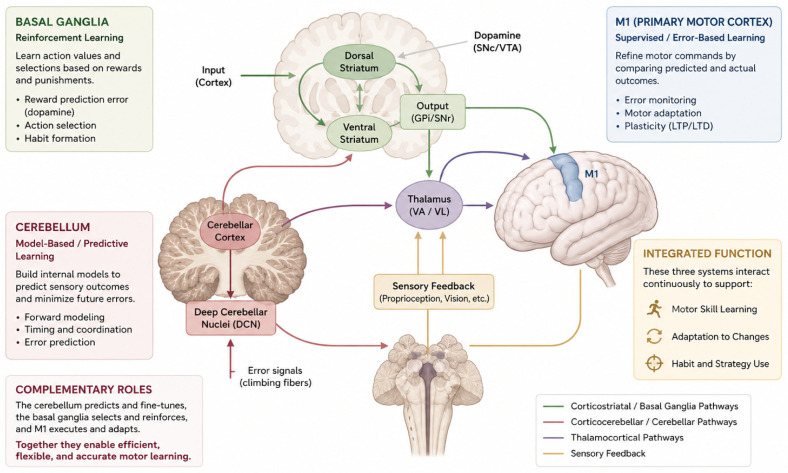
Anatomical location and interconnection diagram of the three core learning mechanisms (cerebellum–basal ganglia–M1).

Before exploring dynamic network reorganization, it is essential to review the core structures and circuits that constitute the motor system. These classical descriptions, extensively covered in foundational textbooks, serve as the anatomical and physiological basis for dynamic models (Figure 1).

### 3.1. From Motor Unit to Muscle: The Periphery

At the most elementary level, movement depends on the motor unit: a single alpha motor neuron in the ventral horn of the spinal cord and all the muscle fibers it innervates. Muscle fibers are classified into Type I (slow-twitch, oxidative, tonic) and Type II (fast-twitch, glycolytic, phasic). Sport training differentially recruits these fiber types: endurance sports predominantly engage Type I fibers, while power sports (sprinting, weightlifting) recruit Type II fibers. The gamma motor system regulates spindle sensitivity, providing crucial feedback for tonic postural maintenance, which is essential in sports requiring stable body positions (e.g., archery, shooting, gymnastics) [14,15].

### 3.2. Spinal and Brainstem Reflexes: The Automatic Foundation

The spinal cord contains reflex arcs that enable rapid, automatic responses [16]. The stretch reflex (monosynaptic), exemplified by the patellar reflex, involves group Ia afferent fibers, the primary sensory afferents of muscle spindles, which synapse directly onto alpha motor neurons [17]. While often studied in isolation, in sport, these reflexes are continuously modulated by supraspinal centers. For example, the muscle tone that maintains posture in a ski jumper during flight or a surfer on a wave is sustained by a combination of spinal reflexes and descending facilitatory inputs from the brainstem reticular formation and vestibular nuclei [18,19].

### 3.3. The Cortex: Motor Maps and the Homunculus

The primary motor cortex (M1, Brodmann area 4), located in the precentral gyrus, is classically considered the executive center for voluntary movement. The somatotopic organization of the motor homunculus reveals that body parts are represented not proportionally to their size but to the richness of their motor innervation. The hands, fingers, and face occupy disproportionately large cortical territories, reflecting the need for fine, independent control [20].

Crucially for sport, these maps are not static. In string players, the cortical representation of the left-hand fingers is expanded compared to non-musicians [21]. In elite athletes, sport-specific cortical reorganization has been documented in tennis players [22], baseball players [23], karateka [24], and basketball players [25]. This use-dependent plasticity is the neural substrate of skill acquisition. Adjacent areas, the supplementary motor area (SMA) and premotor cortex (PMC), are involved in motor planning, sequencing, and the selection of actions based on sensory signals, all critical for complex sport movements [26].

### 3.4. The Corticospinal (Pyramidal) and Extrapyramidal Tracts

Descending motor commands travel via two major pathways. The corticospinal tract originates from pyramidal neurons (including Betz cells) in layer V of M1, decussates at the medullary pyramids, and synapses directly or indirectly onto spinal motor neurons. It is essential for fractionated, fine movements of the distal limbs, the precise finger control required for a basketball free throw or a climber’s grip [27,28].

The extrapyramidal tracts (rubrospinal, reticulospinal, vestibulospinal, tectospinal) originate from brainstem nuclei and provide indirect, parallel control over proximal muscles, posture, and gross movements [29]. In sport, the extrapyramidal system maintains postural stability during dynamic actions: a soccer player kicking a ball requires proximal stability (extrapyramidal) to allow distal precision (pyramidal) [30]. The parallel organization of these systems allows for partial compensation when one system is compromised, a concept with implications for injury rehabilitation.

### 3.5. The Cerebellum: The Feed-Forward Controller

Although constituting only 10% of brain volume, the cerebellum contains more than half of all neurons. Functionally, it is organized into three distinct zones essential for athletic performance: the vestibulo-cerebellum (flocculonodular lobe) controls balance and eye movements; the spino-cerebellum (vermis and intermediate hemisphere) receives somatosensory and spinal inputs to regulate axial and distal musculature; and the cerebro-cerebellum (lateral hemispheres) processes inputs from associative cortical areas via the pontine nuclei, contributing to motor planning and the mental rehearsal of actions, a technique widely used by athletes [31].

At the microcircuit level, the uniform cerebellar cortex comprises three layers (molecular, Purkinje, and granular) and five principal cell types: excitatory granule cells and inhibitory Purkinje, Golgi, stellate, and basket cells [32]. The cerebellum integrates information through two main afferent pathways. Mossy fibers (originating from the spinal cord, brainstem, and cortex) excite granule cells, whose axons bifurcate into parallel fibers that contact the dendritic arbor of Purkinje cells in the molecular layer. In contrast, climbing fibers (exclusively from the inferior olive) wrap around Purkinje cell dendrites, providing a powerful, all-or-none error signal.

This excitatory drive is finely tuned by a local inhibitory network. Basket cells mainly inhibit the soma and initial axon segment of Purkinje cells, while stellate cells primarily inhibit their dendritic domains. This precise inhibitory control sharpens the timing and gains of cerebellar output, allowing Purkinje cells, the sole gamma-aminobutyric acid (GABAergic) output of the cerebellar cortex, to project filtered inhibitory signals to the deep cerebellar nuclei [32,33].

Functionally, this unique architecture enables the cerebellum to act as a feed-forward controller. It constantly compares the voluntary movement (internal forward model) with actual sensory feedback, computes prediction errors, and generates preemptive corrections [33,34,35]. For the athlete, this means that with practice, the cerebellar microcircuit learns to anticipate the consequences of a movement and correct it before sensory feedback even arrives, forming the neurophysiological basis of progressive skill refinement and “muscle memory” [36].

### 3.6. The Basal Ganglia: Action Selection and Habit Formation

The basal ganglia comprise the striatum (caudate, putamen), globus pallidus (external and internal segments), subthalamic nucleus, and substantia nigra (pars compacta and pars reticulata). The classical model distinguishes two pathways: the direct pathway (striatal D1 neurons → internal globus pallidus/substantia nigra pars reticulata (GPi/SNr) → thalamus → cortex), which facilitates movement, and the indirect pathway (striatal D2 neurons → external globus pallidus (GPe) → subthalamic nucleus (STN) → GPi/SNr → thalamus → cortex), which suppresses competing movements. Dopaminergic projections from SNc (pars compacta) modulate these pathways: dopamine D1 receptors (D1) are excitatory (facilitating the direct pathway), while dopamine D2 receptors (D2) are inhibitory (reducing the indirect pathway) [37].

This functional architecture enables the basal ganglia to select and reinforce appropriate motor programs while inhibiting unwanted alternatives. In sport, this mechanism allows an athlete to suppress a habitual but inappropriate response (e.g., a basketball player not dribbling when a pass is the better option) and to automate a well-learned sequence (e.g., a gymnast’s routine). The basal ganglia are central to habit formation and procedural learning, the gradual transition from goal-directed, effortful movement to automatic, fluid execution [38,39].

## 4. The Dynamic Challenge: Why Linear Models Fail to Explain Athletic Skill

The classical model, despite its explanatory power, suffers from a fundamental limitation when applied to sport: it is essentially static and hierarchical [40]. It implies that the cortex plans, the cerebellum corrects, the basal ganglia select, and the spinal cord executes. However, this linear view cannot explain three key observations relevant to sport:

1. Lesion paradox: A cerebellar patient can still perform many voluntary movements, but cannot adapt to a new tennis racket’s weight. A Parkinson’s patient (basal ganglia dopamine depletion) can adapt to a perturbation but cannot form a stable habit. This suggests specialization without isolation [41].
2. Temporal shifts in neural activity: Neuroimaging and transcranial magnetic stimulation (TMS) studies of athletes learning a new skill reveal that the same network (cerebello-thalamo-cortical) is engaged across all learning phases, but the pattern and strength of connectivity change dramatically over time [6,42,43].
3. Multiple simultaneous learning processes: A tennis player learning a new serve simultaneously uses error feedback (ball trajectory), reward signals (successful landing in the box), and mere repetition (use-dependent plasticity). These processes can cooperate or compete, and their relative weighting changes with practice [10,44].

These observations have led to a paradigm shift: motor learning is an emergent property of dynamic, large-scale network reorganization rather than a simple transfer of control from one area to another [8,9]. For the sports scientist, this means that different training strategies are needed at different stages of skill acquisition.

## 5. Core Mechanisms of Motor Learning in Sport

### 5.1. Error-Based Learning: The Cerebellar Coach

Error-based learning (also called supervised learning or sensorimotor adaptation) is the process by which the nervous system compares the expected sensory consequences of a movement with actual feedback and adjusts motor commands to reduce the discrepancy [45,46]. In sport, this is what happens when a basketball player adjusts their free throw based on seeing the ball hit the front rim, or when a swimmer modifies stroke technique based on coach feedback.

Neural substrate: The cerebellum is the master node for error-based learning. The Marr–Albus–Ito theory explains the underlying synaptic mechanism. Climbing fibers from the inferior olive carry an error signal. When a Purkinje cell receives simultaneous input from a climbing fiber (error) and a parallel fiber (context), the synapse between the parallel fiber and the Purkinje cell undergoes long-term depression (LTD) [34,47]. This LTD reduces the inhibitory output of the Purkinje cell onto the deep cerebellar nuclei, disinhibiting the nuclei and allowing a corrective change in motor output. Importantly, this plastic change does not occur in isolation but is embedded within a local inhibitory network. Stellate and basket cells in the molecular layer, together with Golgi cells in the granular layer, regulate the flow of information from mossy fibers, granule cells, and parallel fibers toward Purkinje cells. By shaping the excitability of Purkinje neurons, these inhibitory interneurons determine how strongly contextual information carried by parallel fibers is integrated with the error signal conveyed by climbing fibers. For sport performance, this balance is crucial: precise inhibition allows the cerebellum to filter irrelevant sensory noise, stabilize correct motor patterns, and update movement commands only when an error signal is behaviorally meaningful [32,33,34,47]. Ultimately, this learning is feed-forward: after repeated exposure, the cerebellum learns to predict the error and preemptively correct it, even before sensory feedback arrives [48].

Sport application: Error-based learning is dominant in the early stages of skill acquisition. The novice golfer receives explicit feedback (“your club face was open at impact”) and makes large, conscious corrections. Coaches should provide frequent, precise, immediate feedback during this phase, using video analysis, augmented feedback, and clear error demonstrations [49].

Empirical evidence in sport: Studies using TMS have shown that disrupting cerebellar function impairs visuomotor adaptation in tasks mimicking sport demands [50]. Moreover, successful adaptation is associated with a reduction in cerebellar-brain inhibition (CBI), a measure of the inhibitory influence of the cerebellum over M1, suggesting that LTD-like mechanisms operate in vivo during sport skill learning [6].

### 5.2. Reinforcement Learning: The Striatal Selector

Reinforcement learning (RL) involves learning to select actions that maximize reward (or minimize punishment) based on outcome feedback, without necessarily knowing the correct action [51]. Unlike error-based learning, which relies on sensory prediction errors, RL relies on reward prediction errors (RPE), the difference between obtained and expected reward [52,53]. In sport, this is what happens when a player discovers that a particular grip or stance “feels good” because it leads to a successful outcome, even if they cannot explain why.

The basal ganglia, particularly the striatum, are central to RL. Dopaminergic neurons in the SNc and ventral tegmental area (VTA) phasically fire to unexpected rewards (positive RPE) and dip below baseline to omitted rewards (negative RPE). Dopamine modulates corticostriatal synapses via D1 and D2 receptors. Through repeated reinforcement, successful action sequences become automated habits, represented within cortico-striatal loops [38,54].

Reinforcement learning becomes increasingly important during the intermediate and late stages of learning. Once the athlete can perform the movement reasonably well, explicit error feedback can be reduced and replaced with outcome-based reinforcement (“that serve was in the box, good”). Reward shaping (e.g., scoring systems, positive feedback from coaches) should be emphasized. The transition from error-based to reinforcement-based learning marks the shift from explicit to implicit skill, which is relevant in many sports, including football [55].

Pharmacological blockade of dopamine receptors in M1 impairs motor skill acquisition [56]. Patients with Parkinson’s disease (striatal dopamine depletion) exhibit deficits in reinforcement-based learning while retaining error-based adaptation [57]. In healthy athletes, rewarding feedback enhances retention of motor skills compared to neutral or punishment feedback [53]. This has direct implications for coaching: positive reinforcement should be emphasized once the basic movement pattern is established.

### 5.3. Use-Dependent Learning: The Cortical Sculptor

Use-dependent learning (UDL), also known as use-dependent plasticity, refers to the simple fact that repeatedly performing the same movement changes the neural representation of that movement, making it faster, more stable, and less variable [58,59]. Unlike error-based or reinforcement learning, UDL does not require any explicit performance feedback; mere repetition suffices. In sport, this explains why a gymnast who repeats a routine hundreds of times becomes more consistent, even without constant feedback.

As a neural substrate, M1 is the primary locus of UDL. Repetitive activation of specific corticospinal neurons induces Hebbian plasticity, particularly long-term potentiation (LTP) at synapses within M1 and between M1 and spinal motor neurons [60,61]. This LTP is associated with a transient reduction in GABAergic inhibition, as measured by SICI, allowing for increased excitability and map expansion. With continued training, inhibition is gradually restored to refine and stabilize the new representation [62,63].

UDL is the dominant mechanism in the late stages of learning (automatization). Once the athlete has acquired the basic skill, high-repetition practice consolidates the motor representation. However, a critical nuance: UDL alone can lead to suboptimal solutions if the repeated movement is flawed. A tennis player who practices a technically incorrect serve will simply become more fluent at the flaw [59]. Therefore, UDL should be preceded by adequate error-based learning to establish a correct template.

Simply repeating a thumb movement in a specific direction for 15 min biases subsequent movements in that same direction, even without feedback [60]. Anodal transcranial direct current stimulation (a-tDCS) over M1, which reduces GABA and enhances excitability, improves UDL and motor memory consolidation [64]. However, this excitability-enhancing effect should not be interpreted as a universal target for late-stage automatic performance. While reduced inhibition may facilitate plasticity during skill acquisition or early consolidation, late autonomous performance is often associated with a more refined excitation–inhibition balance and enhanced SICI, which may help suppress competing motor outputs and stabilize highly trained movement representations. Elite musicians and athletes show enhanced SICI, suggesting that long-term training strengthens cortical inhibition to prevent unwanted movement interference [12,65].

### 5.4. Biochemical and Molecular Pathways Underlying Sport-Induced Neuroplasticity

Beyond large-scale neurofunctional circuits, sport skill acquisition depends on a coordinated set of biochemical and molecular pathways that translate repetition, sensory error, reward, metabolic demand, and recovery into long-lasting synaptic and network reorganization. Therefore, the neuroanatomical and neurophysiological mechanisms described above should be interpreted together with the biochemical cascades that regulate synaptic strengthening, synaptic weakening, reward-dependent plasticity, neurotrophic support, and muscle–brain communication.

#### 5.4.1. Glutamatergic Cortical Plasticity

At the cortical level, use-dependent plasticity is mainly supported by glutamatergic mechanisms involving N-methyl-D-aspartate (NMDA) receptor activation, calcium influx, calcium/calmodulin-dependent protein kinase II (CaMKII), protein kinase A (PKA), and protein kinase C (PKC) signaling, α-amino-3-hydroxy-5-methyl-4-isoxazolepropionic acid (AMPA) receptor phosphorylation and trafficking, and cyclic adenosine monophosphate response element-binding protein (CREB)-dependent gene transcription [66,67,68]. Functionally, these molecular events strengthen synapses within motor cortical networks that are repeatedly recruited during training. In sport, this mechanism may explain why high-quality repetition progressively stabilizes task-specific motor patterns, improves movement consistency, and supports the formation of more efficient corticospinal representations. However, because these pathways strengthen what is repeatedly practiced, repetition alone may consolidate both correct and incorrect motor patterns. This reinforces the need for accurate feedback and technical correction before high-volume repetition is emphasized. These cascades provide the molecular basis for long-term potentiation (LTP)-like mechanisms that strengthen task-relevant corticospinal representations during repeated execution of sport-specific movements.

#### 5.4.2. Cerebellar mGluR1–PKC–LTD Pathway

In parallel, cerebellar error-based learning depends on the convergence of climbing fiber error signals and parallel fiber contextual inputs onto Purkinje cells. At the parallel fiber–Purkinje cell synapse, metabotropic glutamate receptor type 1 (mGluR1) activation triggers phospholipase C beta (PLCβ) signaling, inositol 1,4,5-trisphosphate/diacylglycerol (IP3/DAG) production, intracellular calcium release, PKC activation, and AMPA receptor internalization, thereby supporting cerebellar LTD and the updating of internal forward models [69,70]. In the context of sport skill acquisition, this pathway is particularly relevant during the early phases of learning, when movement errors are large and sensory prediction errors are frequent. For example, when a novice athlete repeatedly adjusts a movement based on visual, proprioceptive, or coach-provided feedback, cerebellar LTD-like mechanisms may help recalibrate the relationship between motor commands and their sensory consequences. Thus, the cerebellar mGluR1–PKC–LTD pathway provides a molecular explanation for why precise error feedback is especially important during early skill refinement.

#### 5.4.3. Dopaminergic Corticostriatal Plasticity

Reinforcement learning is shaped by dopaminergic projections from the SNc and VTA to the striatum. Phasic dopamine responses encode reward prediction errors and modulate corticostriatal plasticity via D1- and D2-receptor-dependent signalling pathways involving cyclic adenosine monophosphate (cAMP), protein kinase A (PKA) and the dopamine- and cAMP-regulated phosphoprotein of 32 kDa (DARPP-32). This facilitates the reinforcement of successful motor programmes and the suppression of inefficient or competing actions [71,72,73]. Through these mechanisms, successful motor programs are reinforced, while inefficient or competing actions are progressively suppressed. This is particularly relevant during the associative phase of learning, when the athlete begins to identify which movement solutions produce successful outcomes. In coaching practice, positive reinforcement, reward-based feedback, and outcome-based learning may therefore engage dopaminergic corticostriatal pathways that promote the selection and automatization of effective motor sequences. Over time, repeated reinforcement of successful actions contributes to habit formation and to the transition from effortful control to automatic execution.

#### 5.4.4. BDNF–TrkB Signaling and Growth Factors

A central convergent mechanism across these forms of plasticity is the brain-derived neurotrophic factor (BDNF)–tropomyosin receptor kinase B pathway. Exercise and motor practice increase BDNF availability, which activates tropomyosin receptor kinase B (TrkB) receptors and downstream phosphoinositide 3-kinase (PI3K)–protein kinase B (Akt)–mechanistic target of rapamycin (mTOR), mitogen-activated protein kinase (MAPK)/extracellular signal-regulated kinase (ERK), and phospholipase C gamma (PLCγ)–CaMK–CREB pathways, promoting dendritic spine remodeling, synaptogenesis, local protein synthesis, neuronal survival, and motor memory consolidation [74,75,76]. BDNF–TrkB signaling therefore represents a molecular bridge between repeated motor practice, exercise-induced neuroplasticity, and long-term retention of motor skills. Additional growth factors, including insulin-like growth factor 1 (IGF-1) and vascular endothelial growth factor (VEGF), may further contribute to training-induced neural adaptation by supporting angiogenesis, neurogenesis, synaptic remodeling, and recovery processes [77]. From a sport perspective, these mechanisms suggest that training load, exercise intensity, recovery, and sleep may influence not only performance behavior but also the neurotrophic environment required for consolidation and long-term skill retention.

#### 5.4.5. Muscle–Brain Communication

Importantly, the biochemical effects of sport training are not restricted to the brain. Contracting skeletal muscle releases metabolic and myokine signals that contribute to muscle–brain communication. Lactate, especially during moderate to high-intensity exercise, may act not only as an energetic substrate but also as a signaling molecule capable of increasing BDNF expression through sirtuin 1 (SIRT1)-dependent activation of the peroxisome proliferator-activated receptor gamma coactivator 1-alpha/fibronectin type III domain-containing protein 5 (PGC-1α/FNDC5) pathway [78]. Similarly, exercise-induced PGC-1α/FNDC5/irisin signaling has been proposed as a molecular bridge between peripheral muscular work and central neurotrophic adaptation [77]. Additional systemic mediators, including insulin-like growth factor 1 (IGF-1), VEGF, cathepsin B, anti-inflammatory cytokine modulation, and redox-sensitive pathways, may further regulate angiogenesis, neurogenesis, synaptic remodeling, and recovery after training or injury [79,80,81]. Therefore, sport-induced neuroplasticity should be considered a multilevel process in which neurofunctional pathways provide the anatomical architecture of skill learning, whereas molecular pathways determine the cellular rules by which practice, reward, metabolic stress, and recovery reshape the athletic brain. From a coaching perspective, this implies that training variables such as repetition, feedback timing, reward structure, exercise intensity, sleep, recovery, and environmental specificity may influence not only performance behavior but also the biochemical conditions that enable synaptic consolidation and automatization.

## 6. Integrating the Triad: A Spatiotemporal Model of Network Reorganization for Sport

**Figure 2:**
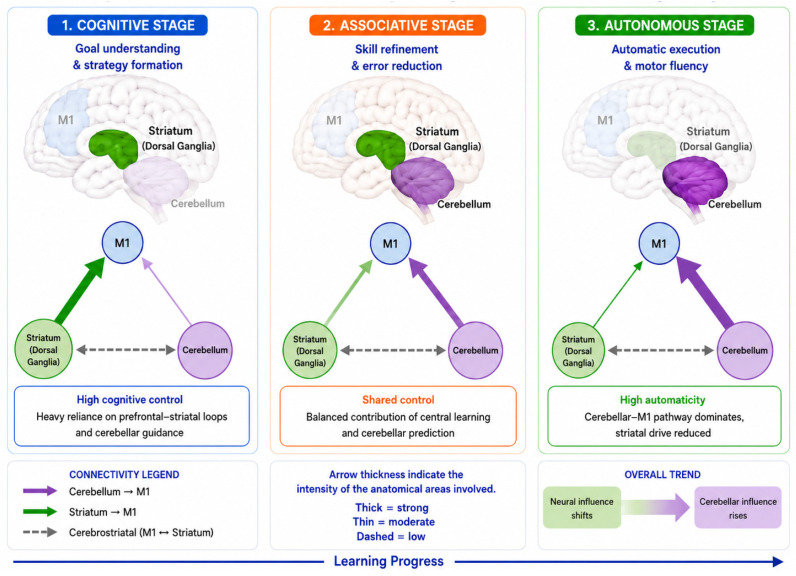
Dynamic shift in cerebellar–M1, striato–M1, and corticostriatal connectivity weights across three stages (cognitive, associative, autonomous).

The three mechanisms described above do not operate in isolation (Figure 2). Rather, they are simultaneously engaged during motor learning, with their relative contributions varying dynamically as a function of learning stage, task demands, and feedback availability [6,11]. We propose a three-stage spatiotemporal model that directly maps onto the phases of sport skill acquisition described in the sport science literature [82,83]. Importantly, these stage-dependent network changes are paralleled by specific biochemical mechanisms. During early learning, large sensory prediction errors preferentially engage cerebellar mGluR1–PKC–LTD signaling, supporting the recalibration of internal forward models [69,70]. During the associative phase, dopaminergic corticostriatal plasticity, involving D1/D2 receptor signaling, cAMP–PKA pathways, and DARPP-32, contributes to reward-based selection and reinforcement of successful motor programs [71,72,73]. During late learning and automatization, repeated execution is increasingly supported by glutamatergic LTP-like mechanisms in motor cortical networks, BDNF–TrkB-dependent synaptic remodeling, growth-factor signaling, and exercise-induced muscle–brain communication, which together promote consolidation, efficiency, and long-term retention [66,67,68,74,75,76,77,79,80,81]. Thus, the proposed model should be interpreted as a multilevel framework in which neural circuits define the functional architecture of learning, whereas biochemical pathways provide the cellular rules that stabilize, weaken, or reinforce motor representations across training stages.

### 6.1. Stage 1: Early Learning (Cognitive/Strategic Phase)

The novice athlete’s movements are slow, clumsy, highly variable, and inefficient, and attention is entirely focused on the task. In this context, the athlete explicitly tries to understand the goal and strategy; for example, a beginner basketball player needs to watch the ball while dribbling and a novice swimmer whose strokes are uncoordinated and exhausting [84].

During early learning, the dorsolateral prefrontal cortex (DLPFC) is highly active, contributing to working memory and strategic planning [85], while the cerebellum is strongly engaged because error signals dominate and each movement yields a large discrepancy between expected and actual sensory feedback. In this phase, LTD within the cerebellar cortex is intense [86]. However, the basal ganglia contribute minimally, as the action policy is not yet formed. M1 is moderately active, but its plasticity is not yet consolidated [87,88].

Accordingly, cerebello-M1 connectivity is strong, reflecting error transmission; cerebello-prefrontal connectivity may facilitate strategic adaptation, while cortico-striatal connectivity is weak [6,42] (Table 1).

Coaching implications:

- Provide frequent, explicit, immediate feedback (verbal, video, augmented).
- Emphasize error identification and correction.
- Use part practice (breaking the skill into components).
- Do not expect automaticity or consistency.
- Keep reinforcement secondary to error correction.

### 6.2. Stage 2: Intermediate Learning (Associative Phase)

In intermediate learning, movements become more fluid, with fewer errors and less variability. The athlete can perform the skill in familiar settings but struggles under pressure or with novelty. Attention demands decrease, but the athlete is still vulnerable to interference [89]. Examples: a golfer who can hit consistently at the driving range but slices under tournament pressure; a swimmer with good technique in practice but who breaks down when fatigued.

Cerebellar activity begins to decline as the internal forward model improves and prediction errors shrink. The basal ganglia become increasingly engaged, as reward feedback (e.g., “that shot felt good”) and success signals begin to select and reinforce the most effective action sequences. M1 undergoes LTP-like plasticity (increased excitability, reduced SICI) to consolidate the motor representation [6,39,89].

The balance shifts, with cerebello-M1 connectivity decreasing (leading to fewer error-driven corrections) and striato-M1 and thalamo-cortical connectivity increasing (facilitating action selection and consolidation). Explicit strategies (prefrontal) are gradually replaced by implicit procedures [54,58,88] (Table 1).

Coaching implications:

- Reduce explicit error feedback frequency.
- Introduce outcome-based feedback (“good shot” vs. specific corrections).
- Use random practice and variable practice to promote generalization.
- Begin to introduce pressure (simulated competition).
- Emphasize positive reinforcement for successful outcomes.

### 6.3. Stage 3: Late Learning (Autonomous Phase)

In late learning, execution becomes automatic, fluid, and efficient. The athlete can perform the skill while attending to other tasks (e.g., a basketball player dribbling while reading the defense). The skill is robust under pressure and resistant to interference. For example, an elite swimmer may execute an effortless stroke even during a maximal sprint, and a tennis player may return serves reflexively [89].

The cerebellum is only minimally active for fine-tuning; the basal ganglia (particularly the putamen) have consolidated the action sequence into a habit. M1 shows enhanced SICI to sharpen the movement representation and suppress competing movements [12,90]. The prefrontal cortex is largely deactivated, freeing cognitive resources for other tasks (e.g., strategy, environmental monitoring) [91].

The network is now efficient and modular. Information flows rapidly through cortico-striatal loops (direct pathway) and M1-spinal connections. Cerebello-M1 connectivity is low but can be rapidly reactivated if an unexpected error occurs. This architecture explains how elite athletes can perform complex skills while attending to other demands [88,89] (Table 1).

Coaching implications:

- Provide minimal explicit feedback (the athlete knows what is correct).
- Use high-repetition, varied practice to maintain and refine.
- Emphasize dual-task training (execute skill while attending to other stimuli) to test and enhance automaticity.
- Train in context-specific environments (e.g., swimmers in water, basketball players on court with defenders).
- Use neurofeedback or brain stimulation (if appropriate) to further enhance inhibition and efficiency.

### 6.4. Neurovascular Coupling and Cerebral Metabolic Support as Modulators of Motor Learning

Although the present model focuses primarily on neural circuits and biochemical pathways involved in sport skill acquisition, these mechanisms depend on adequate neurovascular and metabolic support. Motor learning requires repeated and coordinated activation of cortical, cerebellar, striatal, and spinal networks. Such activation must be accompanied by appropriate increases in regional cerebral blood flow, oxygen delivery, glucose availability, and clearance of metabolic by-products. Therefore, neurovascular coupling should be considered a permissive and modulatory component of motor learning rather than a separate learning mechanism [92,93,94]. This point is particularly relevant because neural activity and hemodynamic responses are not always coupled in a simple one-to-one manner. In conditions characterized by impaired cerebral autoregulation or cerebrovascular dysfunction, such as aging, neurodegenerative disorders, chronic vascular disease, fatigue, or sleep-related breathing disturbances, neural activation may not be adequately matched by local perfusion responses [92,95]. Under these conditions, the same neural input may produce a reduced, delayed, or metabolically inefficient behavioral output. This may partially explain why learning rate, consolidation, fatigue resistance, and automatization differ across age groups and clinical populations. In the context of the proposed three-stage model, neurovascular efficiency may be especially important during the early and intermediate phases of learning, when high attentional demand, error monitoring, and active synaptic remodeling increase metabolic requirements. In late learning, automatization is associated with more efficient and less cognitively demanding motor execution. However, this efficiency still depends on the capacity of the cerebrovascular system to maintain adequate perfusion during prolonged or repeated performance. If neurovascular coupling is impaired, an athlete or patient may show apparently preserved neural recruitment but reduced endurance, poorer consolidation, greater fatigue, or incomplete automatization. Accordingly, sport-induced neuroplasticity should be interpreted as a multilevel process in which neural circuits provide the functional architecture, biochemical pathways regulate synaptic modification, and neurovascular mechanisms provide the metabolic support required for plasticity and sustained performance.

## 7. Sport-Specific Neurophysiological Adaptations: Insights from Elite Athletes

The dynamic network model predicts that long-term, context-specific training should produce measurable neurophysiological adaptations. One of the most elegant demonstrations comes from a study on elite swimmers [12].

### 7.1. The Swimmer Study: Context-Dependent Cortical Inhibition

Sato et al. (2020) [12] studied 14 elite competitive swimmers (with >10 years of training) and 14 age-matched non-swimmer controls. Using TMS, they measured SICI, a GABAergic measure of cortical inhibition in M1, in three conditions: on land (baseline), during water immersion, and on land after immersion.

Elite swimmers demonstrated better sensorimotor performance than non-swimmer controls in the water, whereas no group difference was observed on land. This finding indicates that the behavioral advantage was context-specific, but it should not be interpreted as direct evidence that superior sensorimotor skills were caused by swimming training alone.

Swimmers exhibited increased SICI (greater cortical inhibition) during water immersion compared to land. This increase was absent in non-swimmers.

The enhancement was observed only in the aquatic environment, not on land, indicating that the neural adaptation is functionally tuned to the training context.

The magnitude of SICI enhancement was correlated with years of competitive swimming experience. Although this association is consistent with long-term sport-related adaptation, the cross-sectional design of the study does not allow causal conclusions, and pre-existing neurophysiological differences, self-selection, or other confounding factors cannot be excluded.

Increased SICI reflects sharper, more efficient motor representations. The motor cortex learns to suppress irrelevant neural activity, allowing the desired movement to be executed without interference from competing motor programs. This may represent a neurophysiological correlate of automatization. The fact that this pattern was context-dependent, being observed in water but not on land, suggests that inhibitory cortical states may be tuned to the performance environment. For the swimmer, the aquatic environment may therefore act as a contextual cue associated with a more efficient motor inhibitory state.

Training should occur in the performance environment whenever possible. A basketball player who only practices in an empty gym may not develop the context-dependent inhibition needed to perform in a noisy arena. Swimmers should spend adequate time in the water, not just on dry-land training. Moreover, simulation training (e.g., practicing with crowd noise, competition lighting) may help generalize contextual adaptations [12,96].

### 7.2. The Role of Context and Environment

The swimmer study demonstrates that cortical inhibition is context-dependent [12]. This has practical implications:

- Train in the performance environment whenever possible (water for swimmers, court for tennis players, field for soccer players).
- Use environmental enrichment to strengthen context-specific plasticity (e.g., crowd noise simulators, competition lighting, unfamiliar venues during practice).
- Be aware of contextual interference: a skill learned in one context (e.g., a quiet gym) may not transfer perfectly to another (e.g., a noisy arena). Gradual exposure to target contexts during training is beneficial [97].

### 7.3. Potential for Neuromodulatory Interventions in Sport

While still experimental, non-invasive brain stimulation techniques (a-tDCS, TMS) have shown promise for enhancing motor learning [98,99]. The dynamic model suggests timing matters (Table 2).

Use of brain stimulation in sport is controversial (ethical and safety concerns). However, understanding these mechanisms may inform non-invasive behavioral strategies that mimic stimulation effects (e.g., specific feedback schedules to engage targeted networks) [100]. Importantly, the optimal neuromodulatory target may differ across learning stages. Excitability-enhancing protocols, such as anodal M1 tDCS, may facilitate acquisition or early consolidation, whereas late autonomous performance may require preservation or fine-tuning of inhibitory control, including SICI, to maintain movement selectivity and suppress competing motor programs.

## 8. Implications for Coaching, Training Periodization, and Neurorehabilitation

### 8.1. Phase-Specific Coaching Strategies

The dynamic model provides a neurophysiological rationale for periodizing training content. Coaches should not use the same instructional approach across all phases of learning [101] (Table 3).

### 8.2. Training Periodization Informed by Neural Phase Transitions

Traditional periodization (macro-, meso-, and microcycles) is based on physiological parameters (load, volume, intensity) [102]. The dynamic network model suggests adding a neural periodization dimension:

(1) Early season (preparatory phase): Emphasize error-based learning. Use explicit instruction, video feedback, and blocked practice to establish correct movement templates. Cerebellar engagement is high; coaches should provide frequent, specific corrections.
(2) Mid-season (competition preparation): Emphasize reinforcement learning and emerging use-dependent plasticity. Reduce explicit feedback; increase positive reinforcement; introduce variable and random practice. Begin to simulate competition pressure.
(3) Late season/peak (competition phase): Emphasize use-dependent learning and contextual tuning. High-repetition, sport-specific environments, dual-task training. Minimal explicit feedback. The goal is automation and robustness [103].

### 8.3. Implications for Injury Rehabilitation and Return to Sport

When an athlete is injured, the motor representation of the affected movement may degrade. The dynamic model suggests:

- Early rehabilitation should emphasize error-based learning (explicit feedback, augmented reality, visual feedback) to re-establish accurate movement representations.
- Late rehabilitation should emphasize use-dependent learning (high-repetition) and contextual training (gradual return to sport-specific environments) to restore automatization.
- Clinicians should be aware that motor memory is context-dependent; a movement learned in a clinic may not automatically transfer to the field or court. Gradual, staged exposure is necessary [104].

## 9. Future Directions and Open Questions for Sport Neuroscience

Despite significant progress, several key questions remain unresolved for sport scientists:

- How are multiple plasticity sites coordinated during real-world sport training? Most studies use simple laboratory tasks. Ecologically valid studies tracking elite athletes across a full season are needed. Portable neuroimaging and neurophysiological methods, such as functional near-infrared spectroscopy (fNIRS), portable electroencephalography (EEG), and TMS, are needed [105]. In this context, pairing mobile units (fNIRS plus EEG) represents an optimal approach to disentangle whether suboptimal learning reflects altered neural recruitment, inadequate hemodynamic responses, or a mismatch between neural activation and neurovascular-metabolic support.
- What is the optimal schedule for transitioning from error-based to reinforcement-based learning? Is it better to stay longer in the error-based phase to establish a perfect template, or to transition earlier to promote implicit learning? Individual differences likely matter [106].
- Can we predict which athletes will respond best to which training strategies? Baseline measures of SICI, cerebellar excitability, or dopamine-related genes, such as catechol-O-methyltransferase (COMT) polymorphism, might predict learning trajectories. This could enable personalized training protocols [107].
- How does fatigue (mental or physical) affect the dynamic network reorganization? Does fatigue selectively impair one learning mechanism over others? For example, mental fatigue might impair prefrontal-dependent strategic learning (Stage 1) while sparing cerebellar error-based learning [108].
- What is the role of sleep and offline consolidation in sport skill learning? Sleep is known to consolidate motor memories. Does sleep preferentially consolidate one type of learning (e.g., reinforcement-based habits) over another (error-based adaptation)? [109].
- Can we accelerate the transition from Stage 1 to Stage 3 using targeted interventions? Closed-loop neurofeedback, such as real-time EEG or functional magnetic resonance imaging (fMRI) biofeedback, might allow athletes to consciously modulate their brain state to facilitate plasticity [110].

Advanced techniques such as dual-site TMS (stimulating two brain regions and measuring their interaction), high-density EEG with source localization, and computational modeling will be essential to address these questions in sport contexts [11,46]. Future studies should combine mobile electroencephalography (EEG) and functional near-infrared spectroscopy (fNIRS) during ecologically valid sport tasks to determine whether poor learning, reduced automatization, or fatigue-related performance decline reflects altered neural recruitment, inadequate hemodynamic responses, or a mismatch between neural activation and neurovascular-metabolic support [92,93,94,95]. Future research should also examine whether sleep-related breathing disturbances, such as snoring or obstructive sleep apnea, impair motor memory consolidation and skill automatization through intermittent hypoxia, altered cerebral perfusion, sleep fragmentation, and reduced sleep-dependent plasticity [109,111]. Snoring and sleep apnea are linked to cerebral hypoperfusion, which is a known risk factor for Alzheimer’s disease; this could also negatively affect memory consolidation and, hence, motor development [112].

## 10. Conclusions

Motor learning is not a linear cascade from higher to lower centers, nor a simple transfer of control across a fixed hierarchy. Instead, it is an emergent property of dynamic, large-scale network reorganization involving the cerebellum, basal ganglia, primary motor cortex, and their interconnected loops [113]. The three core learning processes—error-based learning (cerebellar), reinforcement learning (striatal), and use-dependent learning (cortical)—operate in parallel from the very first trial, but their relative weights shift dramatically across the stages of skill acquisition [114].

At the molecular level, these learning processes are supported by partially distinct but interacting biochemical pathways: cerebellar mGluR1–PKC–LTD mechanisms for error-based updating, dopaminergic corticostriatal signaling for reinforcement learning, and glutamatergic LTP-like plasticity, AMPA receptor trafficking, BDNF–TrkB signaling, and growth-factor-mediated remodeling for use-dependent consolidation. In addition, exercise-induced muscle–brain communication through lactate, irisin, IGF-1, VEGF, cathepsin B, inflammatory modulation, and redox-sensitive pathways may create a systemic biochemical environment that facilitates consolidation, recovery, and automatization [66,69,70,71,73,74,75,77,78,80]. Therefore, sport-induced neuroplasticity should be considered a multilevel process in which neural circuits provide the functional architecture, whereas biochemical pathways determine how practice, sensory error, reward, metabolic stress, sleep, and recovery are translated into stable motor memories.

For the athlete and coach, this means that early learning is dominated by the cerebellum and prefrontal cortex [6,57]. Coaches should provide frequent, explicit, error-focused feedback. This is not the time for positive-only reinforcement or high-repetition, mindless practice. Intermediate learning engages the basal ganglia and initiates M1 plasticity [115]. Coaches should reduce explicit feedback, introduce outcome-based reinforcement, and use variable/random practice. Late learning is characterized by reduced cerebellar engagement, enhanced SICI, and efficient cortico-striatal loops [116]. Coaches should use high-repetition, context-specific training, dual-task practice, and minimal explicit instruction.

Moreover, the athlete’s brain is context-sensitive. Elite swimmers show enhanced SICI only in water [12]. This means that training should occur in the performance environment whenever possible, and that context-specific adaptations must be deliberately cultivated [97].

This dynamic, network-level perspective transcends the static homunculus and offers a richer, more clinically and practically relevant framework for sport science. It explains why different athletes need different coaching approaches, why training context matters, and why optimal training strategies must be stage-specific. Future research should focus on tracking network interactions in real-world athletic settings and on developing targeted behavioral, and potentially neuromodulatory, interventions that respect the spatiotemporal dynamics of multisite plasticity [117].

Ultimately, understanding how the brain orchestrates the symphony of learning processes will allow coaches, sport scientists, and clinicians to design more effective training, rehabilitation, and performance optimization strategies [118]. The elite athlete’s brain should not be regarded merely as a well-trained biological system, but as a continuously adapting neural network, progressively shaped by error signals, reward-based reinforcement, and repeated practice, and finely tuned to the specific demands of the sport and its performance environment [13].

## Figures and Tables

**Table 1:** Summary of the Three-Stage Spatiotemporal Model for Sport Skill Acquisition.

Feature	Stage 1: Early (Cognitive Phase)	Stage 2: Intermediate (Associative Phase)	Stage 3: Late (Autonomous Phase)
Athletic behavior	Slow, clumsy, variable, high attention	Fluid, consistent in familiar settings	Automatic, fast, efficient, robust
Dominant learning mechanism	Error-based (cerebellar)	Reinforcement (striatal) + emerging UDL	Use-dependent (cortical) + habit (striatal)
Dominant biochemical drivers	Cerebellar mGluR1–PKC–LTD; error-dependent synaptic weakening	Dopamine, D1/D2 signaling, cAMP–PKA–DARPP-32; BDNF-mediated consolidation	Glutamatergic LTP-like plasticity; BDNF–TrkB; growth factors; muscle–brain myokine/metabolic signaling
Primary neural substrate	Cerebellum + Prefrontal cortex	Basal ganglia + M1	M1 (with enhanced SICI) + putamen
Cortical excitability	Low-moderate	High (LTP-like, reduced SICI)	Refined excitation–inhibition balance with enhanced SICI
Cerebello-M1 connectivity	High (error transmission)	Declining	Low (only for fine tuning)
Striato-M1 connectivity	Low	Increasing	High (habit execution)
Dopamine involvement	Minimal	High (reward signaling)	Tonic (maintenance)
Coach focus	Explicit feedback, error correction, part practice	Outcome feedback, positive reinforcement, variable practice	Repetition, context-specific training, dual-task practice
Example sport task	Learning to coordinate a golf swing	Hitting consistently at driving range	Automatic swing under tournament pressure

**Table 2:** Timing in the Dynamic Model of Non-Invasive Brain Stimulation Techniques.

Timing	Target	Proposed Mechanism	Potential Application
Early learning	Cerebellum (a-tDCS/iTBS)	Enhance LTD-like plasticity, accelerate error-based adaptation	Accelerate initial skill acquisition (e.g., learning a new golf swing)
Late learning/automatization	M1, individualized neuromodulation	Fine-tune excitation–inhibition balance and preserve SICI	Optimize automatic execution and motor efficiency
Pre-competition	M1 (cathodal tDCS or specific TMS protocols)	Modulate SICI, optimize cortical inhibition	“Prime” the motor system for automatic performance

**Table 3:** Coach’s Instructional Approach Proposal Across the Phases of Learning.

Phase	Recommended Coaching Strategies	Avoid
**Stage 1** **(Cognitive)**	● Frequent explicit feedback; ● Video analysis, error correction; ● Part practice; ● Clear demonstrations.	● Positive-only reinforcement without error correction; ● Competition pressure; ● High-repetition of flawed movements.
**Stage 2** **(Associative)**	● Outcome-based feedback; ● Positive reinforcement; ● Variable practice; ● Random practice; ● Simulated pressure.	● Overloading with explicit instructions; ● Insufficient repetition; ● Complete removal of feedback.
**Stage 3** **(Autonomous)**	● Minimal explicit feedback; ● High-repetition varied practice; ● Dual-task training; ● Context-specific training; ● Competition simulation.	● Over-coaching; ● Constant verbal correction; ● Training only in sterile environments.

## Data Availability

No new data were created or analyzed in this study.

## Abbreviations

The following abbreviations are used in this manuscript:

AKT	Protein kinase B (PKB)
AMPA	α-amino-3-hydroxy-5-methyl-4-isoxazolepropionic acid
a-tDCS	Anodal transcranial direct current stimulation
BDNF	Brain-derived neurotrophic factor
CaMKII	Calcium/calmodulin-dependent protein kinase II
cAMP	Cyclic adenosine monophosphate
CBI	Cerebellar-brain inhibition
COMT	Catechol-O-methyltransferase
CREB	Cyclic adenosine monophosphate response element-binding protein
DAG	Diacylglycerol
DARPP-32	Adenosine monophosphate-regulated phosphoprotein of 32 kDa
D1	Dopamine D1 receptor
D2	Dopamine D2 receptor
DLPFC	Dorsolateral prefrontal cortex
EEG	Electroencephalography
ERK	Extracellular signal-regulated kinase
fMRI	Functional magnetic resonance imaging
fNIRS	Functional near-infrared spectroscopy
FNDC5	Fibronectin type III domain-containing protein 5
GABA	Gamma-aminobutyric acid
GPe	External globus pallidus
GPi	Internal globus pallidus
IGF-1	Insulin-like growth factor 1
IP3	Inositol 1,4,5-trisphosphate
iTBS	Intermittent theta-burst stimulation
LTD	Long-term depression
LTP	Long-term potentiation
MAPK	Mitogen-activated protein kinase
M1	Primary motor cortex
mGluR1	Metabotropic glutamate receptor type 1
mTOR	Mechanistic target of rapamycin
NMDA	N-methyl-D-aspartate
PFC	Prefrontal cortex
PGC-1α	Peroxisome proliferator-activated receptor gamma coactivator 1-alpha
PI3K	Phosphoinositide 3-kinase
PKA	Protein kinase A
PKC	Protein kinase C
PLCβ	Phospholipase C beta
PLCγ	Phospholipase C gamma
PMC	Premotor cortex
RL	Reinforcement learning
RPE	Reward prediction error
SICI	Short-interval intracortical inhibition
SIRT1	Sirtuin 1
SMA	Supplementary motor area
SNc	Substantia nigra compacta
SNr	Substantia nigra pars reticulata
STN	Subthalamic nucleus
tDCS	Transcranial direct current stimulation
TrkB	Tropomyosin receptor kinase B
TMS	Transcranial magnetic stimulation
UDL	Use-dependent learning
VEGF	Vascular endothelial growth factor
VTA	Ventral tegmental area

## References

[1] El-Gazzar H.A.E.-M. (2025). Neural Effects of the Repetitions Process during the Athletic Training Program on the Hippocampus and Prefrontal Cortex of the Brain. J. Appl. Sports Sci..

[2] Penfield W., Boldrey E. (1937). Somatic motor and sensory representation in the cerebral cortex of man as studied by electrical stimulation. Brain.

[3] Wiesendanger M. (2010). The pyramidal tract: Recent investigations on its morphology and function. Reviews of Physiology, Biochemmistry and Experimental Pharmacology.

[4] Flash T., Sejnowski T.J. (2001). Computational approaches to motor control. Curr. Opin. Neurobiol..

[5] de Oliveira-Souza R. (2012). The human extrapyramidal system. Med. Hypotheses.

[6] Spampinato D., Celnik P. (2021). Multiple Motor Learning Processes in Humans: Defining Their Neurophysiological Bases. Neuroscientist.

[7] Russo M., Maselli A., Sternad D., Pezzulo G. (2025). Predictive strategies for the control of complex motor skills: Recent insights into individual and joint actions. Curr. Opin. Behav. Sci..

[8] Cocchi L., Zalesky A., Fornito A., Mattingley J.B. (2013). Dynamic cooperation and competition between brain systems during cognitive control. Trends Cogn. Sci..

[9] Constantinidis C., Ahmed A.A., Wallis J.D., Batista A.P. (2023). Common Mechanisms of Learning in Motor and Cognitive Systems. J. Neurosci..

[10] Tsay J.S., Kim H., McDougle S.D., A Taylor J., Haith A., Avraham G., Krakauer J.W., Collins A.G., Ivry R.B. (2024). Fundamental processes in sensorimotor learning: Reasoning, refinement, and retrieval. eLife.

[11] Baladron J., Vitay J., Fietzek T., Hamker F.H. (2023). Correction: The contribution of the basal ganglia and cerebellum to motor learning: A neuro-computational approach. PLoS Comput. Biol..

[12] Sato D., Yamazaki Y., Yamashiro K., Onishi H., Baba Y., Ikarashi K., Maruyama A. (2020). Elite competitive swimmers exhibit higher motor cortical inhibition and superior sensorimotor skills in a water environment. Behav. Brain Res..

[13] Yarrow K., Brown P., Krakauer J.W. (2009). Inside the brain of an elite athlete: The neural processes that support high achievement in sports. Nat. Rev. Neurosci..

[14] Stifani N. (2014). Motor neurons and the generation of spinal motor neuron diversity. Front. Cell. Neurosci..

[15] Li S., Zhuang C., Hao M., He X., Marquez J.C., Niu C.M., Lan N. (2015). Coordinated alpha and gamma control of muscles and spindles in movement and posture. Front. Comput. Neurosci..

[16] Chan V., Pisegna J.M., Rosian R.L., Dicarlo S.E. (1996). Construction of a model demonstrating neural pathways and reflex arcs. Am. J. Physiol..

[17] Pierrot-Deseilligny E., Mazevet D. (2000). The monosynaptic reflex: A tool to investigate motor control in humans. Interest and limits. Neurophysiol. Clin..

[18] Clarac F., Cattaert D., Le Ray D. (2000). Central control components of a ‘simple’ stretch reflex. Trends Neurosci..

[19] Viseux F.J.F., Simoneau M., Pamboris G., Sturbois-Nachef N., Bonnet C., Carrasco M.M., Defebvre L., Billot M., Delval A. (2025). The Reticular formation: An integrative network for postural control. Neurophysiol. Clin..

[20] Chouinard P.A., Paus T. (2006). The Primary Motor and Premotor Areas of the Human Cerebral Cortex. Neuroscientist.

[21] Elbert T., Pantev C., Wienbruch C., Rockstroh B., Taub E. (1995). Increased Cortical Representation of the Fingers of the Left Hand in String Players. Science.

[22] Fourkas A.D., Bonavolont V., Avenanti A., Aglioti S.M. (2008). Kinesthetic imagery and tool-specific modulation of corticospinal representations in expert tennis players. Cereb. Cortex.

[23] Yamashiro K., Sato D., Onishi H., Yoshida T., Horiuchi Y., Nakazawa S., Maruyama A. (2013). Skill-specific changes in somatosensory-evoked potentials and reaction times in baseball players. Exp. Brain Res..

[24] Moscatelli F., Messina G., Valenzano A., Monda V., Viggiano A., Messina A., Petito A., Triggiani A.I., Ciliberti M.A.P., Monda M. (2016). Functional assessment of corticospinal system excitability in karate athletes. PLoS ONE.

[25] Tang W., Wang Y., Qi Y., Fang W., Li X., Liu B., Ning J., Du J., Du X. (2025). Neuroplastic differentiation in motor cortex subregions induced by basketball training: A multimodal diffusion MRI investigation. Neuroimage.

[26] Halsband U., Ito N., Tanji J., Freund H.J. (1993). The role of premotor cortex and the supplementary motor area in the temporal control of movement in man. Brain.

[27] Lemon R.N. (2008). Descending pathways in motor control. Annu. Rev. Neurosci..

[28] Hagah A.M., Jawad A., Al-Imari A. (2021). Effect of Corrective Exercises by an Innovative Device According to Some Biomechanical Variables in the Thrust of the Aiming Arm and the Accuracy of the Free Throw Correction for Basketball Players. http://annalsofrscb.ro.

[29] Lee J., Muzio M.R. (2026). Neuroanatomy, Extrapyramidal System. Extrapyramidal System. StatPearls.

[30] Zemková E., Zapletalová L. (2022). The Role of Neuromuscular Control of Postural and Core Stability in Functional Movement and Athlete Performance. Front. Physiol..

[31] Lara-Aparicio S.Y., Laureani-Fierro A., Morgado-Valle C., Beltrán-Parrazal L., Rojas-Durán F., García L., Toledo-Cárdenas R., Hernández M., Manzo J., Pérez C. (2022). Latest research on the anatomy and physiology of the cerebellum. Neurol. Perspect..

[32] D’Angelo E., Mazzarello P., Prestori F., Mapelli J., Solinas S., Lombardo P., Cesana E., Gandolfi D., Congi L. (2011). The cerebellar network: From structure to function and dynamics. Brain Res. Rev..

[33] Uusisaari M., de Schutter E. (2011). The mysterious microcircuitry of the cerebellar nuclei. J. Physiol..

[34] Ito M. (1972). Neural design of the cerebellar motor control system. Brain Res..

[35] Marr D. (1969). A theory of cerebellar cortex. J. Physiol..

[36] Cumming K.T., Reitzner S.M., Hanslien M., Skilnand K., Seynnes O.R., Horwath O., Psilander N., Sundberg C.J., Raastad T. (2024). Muscle memory in humans: Evidence for myonuclear permanence and long-term transcriptional regulation after strength training. J. Physiol..

[37] Lanciego J.L., Luquin N., Obeso J.A. (2012). Functional neuroanatomy of the basal ganglia. Cold Spring Harb. Perspect. Med..

[38] Park J., Coddington L.T., Dudman J.T. (2020). Basal Ganglia Circuits for Action Specification. Annu. Rev. Neurosci..

[39] Grillner S. (2025). How circuits for habits are formed within the basal ganglia. Proc. Natl. Acad. Sci. USA.

[40] Shi J., Wang J., Lang J., Zhang Z., Bi Y., Liu R., Jiang S., Hou L. (2020). Effect of different motor skills training on motor control network in the frontal lobe and basal ganglia. Biol. Sport.

[41] D’Angelo E., Casali S. (2013). Seeking a unified framework for cerebellar function and dysfunction: From circuit operations to cognition. Front. Neural Circuits.

[42] Areshenkoff C.N., de Brouwer A.J., Gale D.J., Nashed J.Y., Smallwood J., Flanagan J.R., Gallivan J.P. (2024). Distinct patterns of connectivity with the motor cortex reflect different components of sensorimotor learning. PLoS Biol..

[43] Moscatelli F., Messina A., Valenzano A., Monda V., Salerno M., Sessa F., La Torre E., Tafuri D., Scarinci A., Perrella M. (2021). Transcranial magnetic stimulation as a tool to investigate motor cortex excitability in sport. Brain Sci..

[44] Nardi F., Faisal A.A., Haar S. (2025). Motor learning mechanisms are not modified by feedback manipulations in a real-world task. npj Sci. Learn..

[45] Streng M.L., Popa L.S., Ebner T.J. (2022). Cerebellar Representations of Errors and Internal Models. Cerebellum.

[46] Feulner B., Perich M.G., Miller L.E., Clopath C., Gallego J.A. (2025). A neural implementation model of feedback-based motor learning. Nat. Commun..

[47] Hansel C., Linden D.J., D’Angelo E. (2001). Beyond parallel fiber LTD: The diversity of synaptic and non-synaptic plasticity in the cerebellum. Nat. Neurosci..

[48] Lisberger S.G. (2021). The Rules of Cerebellar Learning: Around the Ito Hypothesis. Neuroscience.

[49] Ferguson C., Collins D., Carson H.J. (2025). Golf coaches’ perceptions of the role and use of player errors in motor learning: An exploratory survey. Phys. Educ. Sport Pedagog..

[50] Schlerf J.E., Galea J.M., Bastian A.J., Celnik P.A. (2012). Dynamic modulation of cerebellar excitability for abrupt, but not gradual, visuomotor adaptation. J. Neurosci..

[51] Sutton R.S., Barto A.G. (1998). Reinforcement Learning: An Introduction.

[52] Izawa J., Shadmehr R. (2011). Learning from sensory and reward prediction errors during motor adaptation. PLoS Comput. Biol..

[53] Galea J.M., Mallia E., Rothwell J., Diedrichsen J. (2015). The dissociable effects of punishment and reward on motor learning. Nat. Neurosci..

[54] Doyon J., Bellec P., Amsel R., Penhune V., Monchi O., Carrier J., Lehéricy S., Benali H. (2009). Contributions of the basal ganglia and functionally related brain structures to motor learning. Behav. Brain Res..

[55] Liu B., Pu Z., Zhang T., Wang H., Yi J., Mi J. (2023). Learning to Play Football from Sports Domain Perspective: A Knowledge-Embedded Deep Reinforcement Learning Framework. IEEE Trans. Games.

[56] Hosp J.A., Luft A.R. (2013). Dopaminergic meso-cortical projections to M1: Role in motor learning and motor cortex plasticity. Front. Neurol..

[57] Doyon J., Penhune V., Ungerleider L.G. (2003). Distinct contribution of the cortico-striatal and cortico-cerebellar systems to motor skill learning. Neuropsychologia.

[58] Mawase F., Lopez D., Celnik P.A., Haith A.M. (2018). Movement Repetition Facilitates Response Preparation. Cell Rep..

[59] Diedrichsen J., White O., Newman D., Lally N. (2010). Use-dependent and error-based learning of motor behaviors. J. Neurosci..

[60] Classen J., Liepert J., Wise S.P., Hallett M., Cohen L.G. (1998). Rapid plasticity of human cortical movement representation induced by practice. J. Neurophysiol..

[61] Rioult-Pedotti M.S., Friedman D., Donoghue J.P. (2000). Learning-induced LTP in neocortex. Science.

[62] Kida H., Mitsushima D. (2018). Mechanisms of motor learning mediated by synaptic plasticity in rat primary motor cortex. Neurosci. Res..

[63] Dai W., Pi Y.L., Ni Z., Tan X.Y., Zhang J., Wu Y. (2016). Maintenance of balance between motor cortical excitation and inhibition after long-term training. Neuroscience.

[64] Galea J.M., Celnik P. (2009). Brain polarization enhances the formation and retention of motor memories. J. Neurophysiol..

[65] Rosenkranz K., Williamon A., Rothwell J.C. (2007). Motorcortical excitability and synaptic plasticity is enhanced in professional musicians. J. Neurosci..

[66] Fritsch B., Reis J., Martinowich K., Schambra H.M., Ji Y., Cohen L.G., Lu B. (2010). Direct current stimulation promotes BDNF-dependent synaptic plasticity: Potential implications for motor learning. Neuron.

[67] Vaynman S., Ying Z., Gomez-Pinilla F. (2004). Hippocampal BDNF mediates the efficacy of exercise on synaptic plasticity and cognition. Eur. J. Neurosci..

[68] Hasan M.T., Hernández-González S., Dogbevia G., Treviño M., Bertocchi I., Gruart A., Delgado-García J.M. (2013). Role of motor cortex NMDA receptors in learning-dependent synaptic plasticity of behaving mice. Nat. Commun..

[69] Kano M., Watanabe T. (2017). Type-1 metabotropic glutamate receptor signaling in cerebellar Purkinje cells in health and disease. F1000Research.

[70] Fiala J.C., Grossberg S., Bullock D. (1996). Metabotropic glutamate receptor activation in cerebellar purkinje cells as substrate for adaptive timing of the classically conditioned eye-blink response. J. Neurosci..

[71] Greengard P., Allen P.B., Nairn A.C. (1999). Beyond the dopamine receptor: The DARPP-32/protein phosphatase-1 cascade. Neuron.

[72] Qian Y., Forssberg H., Heijtz R.D. (2015). Motor skill learning is associated with phase-dependent modifications in the striatal cAMP/PKA/DARPP-32 signaling pathway in rodents. PLoS ONE.

[73] Schultz W. (2016). Dopamine reward prediction error coding. Dialogues Clin. Neurosci..

[74] Yoshii A., Constantine-Paton M. (2010). Postsynaptic BDNF-TrkB signaling in synapse maturation, plasticity, and disease. Dev. Neurobiol..

[75] Kowiański P., Lietzau G., Czuba E., Waśkow M., Steliga A., Moryś J. (2018). BDNF: A Key Factor with Multipotent Impact on Brain Signaling and Synaptic Plasticity. Cell. Mol. Neurobiol..

[76] Schirò G., Iacono S., Ragonese P., Aridon P., Salemi G., Balistreri C.R. (2022). A Brief Overview on BDNF-Trk Pathway in the Nervous System: A Potential Biomarker or Possible Target in Treatment of Multiple Sclerosis?. Front. Neurol..

[77] Wrann C.D., White J.P., Salogiannnis J., Laznik-Bogoslavski D., Wu J., Ma D., Lin J.D., Greenberg M.E., Spiegelman B.M. (2013). Exercise induces hippocampal BDNF through a PGC-1α/FNDC5 pathway. Cell Metab..

[78] El Hayek L., Khalifeh M., Zibara V., Abi Assaad R., Emmanuel N., Karnib N., El-Ghandour R., Nasrallah P., Bilen M., Ibrahim P. (2019). Lactate mediates the effects of exercise on learning and memory through sirt1-dependent activation of hippocampal brain-derived neurotrophic factor (BDNF). J. Neurosci..

[79] Cotman C.W., Berchtold N.C., Christie L.A. (2007). Exercise builds brain health: Key roles of growth factor cascades and inflammation. Trends Neurosci..

[80] Moon H.Y., Becke A., Berron D., Becker B., Sah N., Benoni G., Janke E., Lubejko S.T., Greig N.H., Mattison J.A. (2016). Running-Induced Systemic Cathepsin B Secretion Is Associated with Memory Function. Cell Metab..

[81] Cefis M., Chaney R., Wirtz J., Méloux A., Quirié A., Leger C., Prigent-Tessier A., Garnier P. (2023). Molecular mechanisms underlying physical exercise-induced brain BDNF overproduction. Front. Mol. Neurosci..

[82] Hashizume K., Matsuo T. (2004). Temporal and spatial factors reflecting performance improvement during learning three-ball cascade juggling. Hum. Mov. Sci..

[83] Williams A.M., Ford P.R. (2009). Promoting a skills-based agenda in olympic sports: The role of skill-acquisition specialists. J. Sports Sci..

[84] Marineau E., Ducas J., Mathieu J., Rodriguez A.D.P., Descarreaux M., Abboud J. (2024). From Novice to Expert: How Expertise Shapes Motor Variability in Sports Biomechanics—A Scoping Review. Scand. J. Med. Sci. Sports.

[85] Barbey A.K., Koenigs M., Grafman J. (2013). Dorsolateral prefrontal contributions to human working memory. Cortex.

[86] Popa L.S., Ebner T.J. (2019). Cerebellum, predictions and errors. Front. Cell. Neurosci..

[87] Favila N., Gurney K., Overton P.G. (2024). Role of the basal ganglia in innate and learned behavioural sequences. Rev. Neurosci..

[88] Cereda F. (2024). Addressing complexity in motor learning: Practical strategies for teachers and coaches. Am. J. Creat. Educ..

[89] Taylor J.A., Ivry R.B. (2012). The role of strategies in motor learning. Ann. N. Y. Acad. Sci..

[90] Kida H., Tsuda Y., Ito N., Yamamoto Y., Owada Y., Kamiya Y., Mitsushima D. (2016). Motor Training Promotes Both Synaptic and Intrinsic Plasticity of Layer II/III Pyramidal Neurons in the Primary Motor Cortex. Cereb. Cortex.

[91] Miller E.K. (2000). The prefontral cortex and cognitive control. Nat. Rev. Neurosci..

[92] Iadecola C. (2004). Neurovascular regulation in the normal brain and in Alzheimer’s disease. Nat. Rev. Neurosci..

[93] Girouard H., Iadecola C. (2006). Neurovascular coupling in the normal brain and in hypertension, stroke, and Alzheimer disease. J. Appl. Physiol..

[94] Attwell D., Laughlin S.B. (2001). An energy budget for signaling in the grey matter of the brain. J. Cereb. Blood Flow Metab..

[95] Kisler K., Nelson A.R., Montagne A., Zlokovic B.V. (2017). Cerebral blood flow regulation and neurovascular dysfunction in Alzheimer disease. Nat. Rev. Neurosci..

[96] Dann R., Duhig S., Roberts L., Kelly V., Renshaw I., Headrick J. (2024). A principled approach to skill acquisition in competitive surfing: Embracing representative learning design. Int. J. Sports Sci. Coach..

[97] Wang Y., Lin Y., Ran Q., Cao N., Xia X., Tan X., Wu Y., Zhang J., Liu K., Liu H. (2024). Dorsolateral prefrontal cortex to ipsilateral primary motor cortex intercortical interactions during inhibitory control enhance response inhibition in open-skill athletes. Sci. Rep..

[98] Avery M.C., Krichmar J.L. (2017). Neuromodulatory systems and their interactions: A review of models, theories, and experiments. Front. Neural Circuits.

[99] Wang H., Shi Z., Sun W., Zhang J., Wang J., Shi Y., Yang R., Li C., Chen D., Wu J. (2020). Development of a Non-invasive Deep Brain Stimulator with Precise Positioning and Real-Time Monitoring of Bioimpedance. Front. Neuroinform..

[100] Darby R.R., Pascual-Leone A. (2017). Moral enhancement using non-invasive brain stimulation. Front. Hum. Neurosci..

[101] Otte F.W., Millar S.-K., Klatt S. (2019). Skill Training Periodization in ‘Specialist’ Sports Coaching—An Introduction of the ‘PoST’ Framework for Skill Development. Front. Sports Act. Living.

[102] Naclerio F., Moody J., Chapman M. (2013). Applied periodization: A methodological approach. J. Hum. Sport Exerc..

[103] Stone M.H., Hornsby W.G., Haff G.G., Fry A.C., Suarez D.G., Liu J., Gonzalez-Rave J.M., Pierce K.C. (2021). Periodization and block periodization in sports: Emphasis on strength-power training—A provocative and challenging narrative. J. Strength Cond. Res..

[104] Gokeler A., Neuhaus D., Benjaminse A., Grooms D.R., Baumeister J. (2019). Principles of Motor Learning to Support Neuroplasticity After ACL Injury: Implications for Optimizing Performance and Reducing Risk of Second ACL Injury. Sports Med..

[105] Liu K., Zhu Q., Xu W. (2025). Requirement of a complex motor task to identify neuroplastic changes in motor control of the lower extremity in patients with anterior cruciate ligament reconstruction: A fNIRS study. Front. Hum. Neurosci..

[106] De Brouwer A.J., Areshenkoff C.N., Rashid M.R., Flanagan J.R., Poppenk J., Gallivan J.P. (2022). Human variation in error-based and reinforcement motor learning is associated with entorhinal volume. Cereb. Cortex.

[107] Humińska-Lisowska K. (2024). Dopamine in Sports: A Narrative Review on the Genetic and Epigenetic Factors Shaping Personality and Athletic Performance. Int. J. Mol. Sci..

[108] Qi P., Ru H., Gao L., Zhang X., Zhou T., Tian Y., Thakor N., Bezerianos A., Li J., Sun Y. (2019). Neural Mechanisms of Mental Fatigue Revisited: New Insights from the Brain Connectome. Engineering.

[109] Lugassy D., Herszage J., Pilo R., Brosh T., Censor N. (2018). Consolidation of complex motor skill learning: Evidence for a delayed offline process. Sleep.

[110] Sitaram R., Ros T., Stoeckel L., Haller S., Scharnowski F., Lewis-Peacock J., Weiskopf N., Blefari M.L., Rana M., Oblak E. (2017). Closed-loop brain training: The science of neurofeedback. Nat. Rev. Neurosci..

[111] Yan L., Park H.R., Kezirian E.J., Yook S., Kim J.-H., Joo E.Y., Kim H. (2021). Altered regional cerebral blood flow in obstructive sleep apnea is associated with sleep fragmentation and oxygen desaturation. J. Cereb. Blood Flow Metab..

[112] Andrade A.G., Bubu O.M., Varga A.W., Osorio R.S. (2018). The Relationship between Obstructive Sleep Apnea and Alzheimer’s Disease. J. Alzheimer’s Dis..

[113] Roth R.H., Ding J.B. (2024). Cortico-basal ganglia plasticity in motor learning. Neuron.

[114] Lundbye-Jensen J., Christiansen L. (2025). Skill Acquisition and Motor Learning. Neuroplasticity-Based Neurorehabilitation.

[115] Ungerleider L.G., Doyon J., Karni A. (2002). Imaging brain plasticity during motor skill learning. Neurobiol. Learn. Mem..

[116] Bassett D.S., Yang M., Wymbs N.F., Grafton S.T. (2015). Learning-induced autonomy of sensorimotor systems. Nat. Neurosci..

[117] Dovgan N. (2023). An Interdisciplinary Approach to Training Physical Education and Sports Specialists: Insights from Neurophysiology, Neurobiology, Sports Psychology, and Pedagogy. SSRN Electron. J..

[118] Roy A., Yadav M.K., Dutt R., Singh A., Pal C., Tirkey R., Sharma R., Kumar A., Raj U., Gupta S.S. (2025). From Synapses to Stadiums: How Brain Physiology Informs Sports Training for Optimal Performance and a Sound Mind. Int. J. Sci. Res. Technol..

